# Parainfluenza and corona viruses in a fallow deer (*Dama dama*) with fatal respiratory disease

**DOI:** 10.3389/fvets.2022.1059681

**Published:** 2022-12-06

**Authors:** Akbar Dastjerdi, Tobias Floyd, Vanessa Swinson, Hannah Davies, Andrew Barber, Alan Wight

**Affiliations:** ^1^Animal and Plant Health Agency (APHA)–Weybridge, Addlestone, United Kingdom; ^2^APHA-Thirsk, Thirsk, United Kingdom; ^3^Clevedale Vets, Upleatham Veterinary Surgery, Home Farm, Redcar, United Kingdom; ^4^APHA-Starcross, Exeter, United Kingdom

**Keywords:** fallow deer (*Dama dama*), deer coronavirus, deer parainfluenza virus, deer pneumonia, deer fatality, coronaviruses

## Abstract

Parainfluenza virus type 3 (PIV-3) and coronaviruses (CoV) are commonly found in respiratory tracts of ruminants and capable of causing clinical disease. Here, we investigated the cause of ill-thrift and sudden death in a five-month-old male fallow deer which occurred in December 2019. The calf was one of the five calves in a herd of 170 deer that, along with three adult hinds, died during a 2-week period. The deer calves were in a shed, sharing airspace with young cattle that had been reported to be coughing. Significant gross pathology was observed in the respiratory and alimentary tracts of the deer calf and histopathology of the lung and trachea was suggestive of likely involvement of PIV-3. Strong and specific cytoplasmic labeling of bronchiolar epithelium and terminal airway, alike those seen with PIV-3 pneumonia in cattle, was observed using a polyclonal bovine PIV-3 antibody. Metagenomic analysis detected a PIV-3 and a CoV in the lung tissue. The PIV-3 L protein gene had the highest sequence identity with those of bovine PIV-3 (83.1 to 98.4%) and phylogenetically clustered with bovine PIV-3 in the genotype C. The CoV spike protein gene shared 96.7% to 97.9% sequence identity with those of bovine CoVs, but only 53.1% identity with SARS-CoV-2 reference virus. We believe this is the first report of PIV-3 and CoV co-infection in fallow deer and their association with fatal pneumonia; major pathology caused by PIV-3.

## Introduction

Bovine parainfluenza virus type 3 (BPIV-3) is one of the main pathogens involved in the bovine respiratory disease complex, with potential to increase susceptibility to other respiratory pathogens. In cattle, the manifestation of BPIV-3 infection could range from subclinical to acute respiratory disease, with high fever, a nasal discharge and coughing. Co-infection of BPIV-3 with other viral, bacterial and mycoplasmal pathogens such as bovine respiratory syncytial virus (BRSV), bovine herpesvirus 1 (BoHV-1), *Mycoplasma bovis, Pasteurella multocida* and *Mannheimia haemolytica* are generally common in cattle. The involvement of more than one pathogen is likely to exacerbate respiratory system pathology. BPIV-3 infection has been serologically detected in several domestic and free-ranging ungulates, including cattle, goats, sheep, camels, and new-world camelids (1). Cross-species infection of BPIV-3 has also been documented in sheep (2, 3), water buffalo (4) and humans (5). PIV-3 of unknown genotype has also been isolated from nasal swabs of one healthy fallow deer and one mule deer (6). A live attenuated BPIV-3 vaccine, which was developed to prevent human PIV-3 disease, was shown to be infectious and immunogenic in 6- to 36-month-old infants and children (7). This further emphasizes cross-species potential of BPIV-3. PIV-3 can be transmitted either by aerosol or by contact with fomites contaminated with nasal discharge.

BPIV-3 is an enveloped, negative strand RNA virus in the species *Bovine respirovirus 3*, of the genus *Respirovirus*, within the family *Paramyxoviridae*, order *Mononegavirales* (8, 9), with three genotypes: BPIV-3A, BPIV-3B and BPIV-3C (10, 11). The BPIV-3A genotype has a worldwide prevalence (12), whilst BPIV-3B is restricted to Australia, Argentina, and the USA (4, 10, 13). BPIV-3C was first identified in China, but has also been isolated in Korea, the USA, Argentina, and Turkiye (4, 11, 13–15). A Japanese BPIV-3C isolate (HS9) was found to be distinct from other reported BPIV-3 strains (sequence accession number LC000638.1) (16).

Similar to PIV-3, coronaviruses (CoVs) also infect diverse mammalian and avian species causing respiratory, enteric, neurologic, and hepatic disorders (17). Since identification of SARS-CoV-1 in 2003, a significant increase in the number of emerging CoVs have been recorded (18, 19). Discovery of these new viruses has highlighted the ability of CoVs to jump host-species barriers e.g., SARS-CoV-1 in palm civet and humans (20) and Middle East Respiratory Syndrome CoV in camels and humans (21–23). Naturally acquired infections of SARS-CoV-2 have also been confirmed in pet dogs, cats, ferrets, wild tigers, lions, puma, snow leopard, Western lowland gorillas, farmed American mink and white-tailed deer, *Odocoileus virginianus* [reviewed by (24, 25)]. Human-to-deer transmission events followed by subsequent deer-to-deer spread (26, 27). Bovine CoV (BCoV) represent excellent examples of CoVs that extensively cross species barriers (28, 29). The viruses have been identified in various domestic and wild ruminant species (water buffalo, sheep, goat, dromedary camel, llama, alpaca, deer, wild cattle, antelopes, giraffes, and wild goats) as well as dogs and humans [reviewed by Vlasova and Saif (29)]. Due to the phylogenetic closeness of BCoV and SARS-CoV-2, the use of cow's milk immune to BCoV has even been proposed for control of the virus in humans (30).

BCoVs are enveloped, positive strand RNA viruses in the genus *Betacoronavirus* within the family *Coronaviridae*, order *Nidovirales* (https://talk.ictvonline.org/). Coronaviruses are recently divided into two subfamilies - *Letovirinae* and *Orthocoronavirinae*, each comprising of one, *Alphaletovirus* and four, *Alphacoronavirus, Betacoronavirus, Gammacoronavirus* and *Deltacoronavirus* genera respectively. The first two genera of *Orthocoronavirinae* include only mammalian CoVs, while all avian CoVs are members of the other two genera.

Here, we present diagnostic investigation of loss of body condition, diarrhea, malaise, dyspnoea, and sudden death in a herd of 170 fallow deer which occurred in December 2019. Five calves, aged around 5 months old, and three adult hinds died over a two-week period. The hinds and calves were together at pasture when the first affected deer, an older hind, was found dead. The hinds were deemed to be in poorer body condition than in previous years. At this time the calves (*n* = 50) were weaned, brought indoors and fed silage, whilst the hinds remained at pasture. Over the following 2 weeks, some of the calves began to appear lethargic and stood apart from the group, and one calf developed diarrhea. The calves were treated with injectable ivermectin, and the weaker calves were given a trace element bolus. The calves were housed in a shed sharing a common airspace with a group of heifers but had no direct contact. Coughing was noted in the heifer group, but they were otherwise healthy. None of the animals were vaccinated against respiratory pathogens. No further issues with the deer calves or the heifers were observed. The carcass of one deer calf that died was submitted to APHA-Thirsk Veterinary Investigation Center (VIC) for examination. We carried out gross pathological and histopathological examinations, immunohistochemistry (IHC) and next generation sequencing (NGS) to investigate this disease, characterize the pathogens detected, and provide evidence for their association with the disease.

## Materials and methods

### Post-mortem examination

The post-mortem examination followed the standard protocol used in the APHA VIC, ensuring a systematic approach and comprehensive assessment of all body systems. Charcoal-medium swabs were taken for bacteriology, and a range of fresh and formalin-fixed tissue samples; lung, liver, spleen, kidney, brainstem, urine, aqueous humor, feces and abomasal and small intestinal content were collected and stored for further analysis.

### Pathology and immunohistochemistry (IHC)

Sections (3–5 μm) of paraffin embedded formalin-fixed tissues were either stained with haematoxylin and eosin (H&E) or subjected to IHC for microscopic examination. IHC was performed using rabbit polyclonal antibody against formalin-killed whole BPIV-3 (Prairie Diagnostic Services Incorporated) and monoclonal antibodies (mAb) against BRSV (Vector Laboratories) and BCoV (RTI, LLC). The BCoV mAb is produced as mouse ascites fluid and reacts with North American strains of BCoV, recognizing an epitope on the nucleocapsid protein of the virus. The staining was continued with DAKO REAL EnVision Detection System, Peroxidase/DAB+, Rabbit/Mouse (Agilent Technologies) for visualization.

### Next generation sequencing

Nucleic acid extraction from frozen lung tissue was performed as described by Dastjerdi et al. (31). NGS was carried out at the Central Sequencing Unit, APHA-Weybridge using Small Whole-Genome - Nextera XT (Illumina) kit for library preparation and Illumina NextSeq sequencing platform. Sequence reads were analyzed using SeqMan NGen 17 (DNASTAR) through *de novo* and reference guided assembly applications using GenBank virus reference sequences. Phylogenetic analysis of the PIV-3 and CoV detected in this study were carried out using amino acid sequences for complete L and spike proteins, respectively. The sequences were aligned using the MegAlign software (DNASTAR). Phylogenetic analysis was conducted in MEGA X (32) and the evolutionary history was inferred using the Maximum Likelihood method, Le Gascuel model (33) and 500 bootstrap estimation (34). Initial tree for the heuristic search was obtained automatically by applying Neighbor-Join and BioNJ algorithms to a matrix of pairwise distances estimated using the JTT model, and then selecting the topology with superior log likelihood value.

### Other investigations

Aerobic bacteriology was performed by routine methods using sheep blood agar and MacConkey's agar plates incubated at 37°C for 24 h. *Clostridium perfringens* toxin testing on small intestinal contents was carried out using Enterotoxaemia ELISA kit (Bio-X Diagnostics) as per instructions. PCR for ovine herpesvirus-2 on spleen sample was undertaken as described by Baxter et al. (35).

Endoparasite burden was assessed through a combination of the modified McMaster technique for fecal egg counting (36) and an estimated total worm count undertaken on abomasal and small intestinal content (37).

## Results

### Gross pathology

On gross examination, the submitted carcass was in suboptimal body condition. The main gross findings were limited to the respiratory tract and included diffuse consolidation of the cranial lung lobes with multiple small abscesses. Airway mucosa was reddened and contained stable foam with flecks of pus and adult nematodes that were identified *as Dictyocaulus spp*. Additionally, the abomasal mucosa was reddened and the small intestinal contents were watery in consistency, but the colon contained formed fecal pellets.

Parasitological examination revealed 1050 trichostrongyle-type eggs per gram of feces, with a combined infestation of the abomasum by *Ostertagia spp* and *Trichostrongylus axei. Escherichia coli* (*E. coli*) was isolated in pure growth from the lung and brain. Tests for *C perfringens* toxin and ovine herpesvirus-2 were negative.

### Histopathology

**Figure 1:**
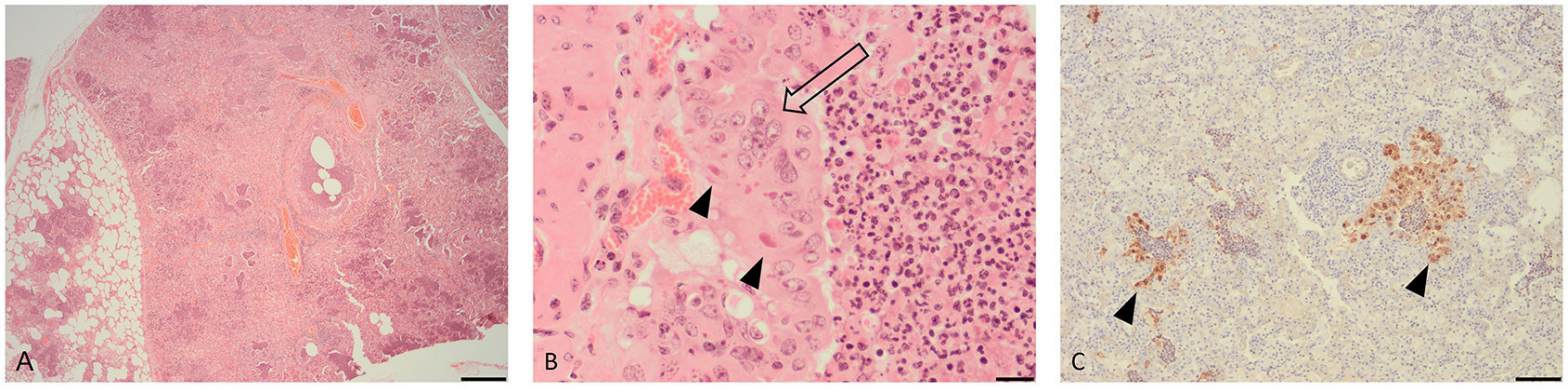
Histopathological changes in the lung of a fallow deer (*Dama dama*). **(A)** Low magnification view of lung showing leukocyte infiltration and collapse of airways and alveoli following a lobular pattern (H&E, bar = 500 μm). **(B)** High magnification of an airway showing hyperplastic bronchiolar epithelium forming multinucleate syncytia (open arrow) with eosinophilic cytoplasmic inclusions (arrow heads) (H&E, bar = 50 μm). **(C)** Immunohistochemistry for parainfluenza virus type 3 showing viral antigen within airway epithelium (arrow heads) (IHC, bar = 100 μm).

Microscopic examination of the lung tissue revealed widespread collapse and leukocyte infiltration of airspaces following a lobular pattern (Figure 1A). There were also nodular foci of lytic necrosis and suppurative inflammation (abscesses) and cross-sections of adult and larval nematodes, consistent with the gross findings. Significantly, bronchioles showed changes characteristic of acute viral infection including epithelial necrosis, attenuation and hyperplasia, with epithelial syncytia and eosinophilic intra-cytoplasmic inclusions (Figure 1B).

### Immunohistochemistry

IHC for BPIV-3 demonstrated viral antigen within the cytoplasm of epithelium in affected bronchioles and alveoli (Figure 1C). In other words, the IHC revealed co-localization of viral antigen with pathology in airways. There was no positive staining for BRSV.

### Sequence analysis

**Figure 2:**
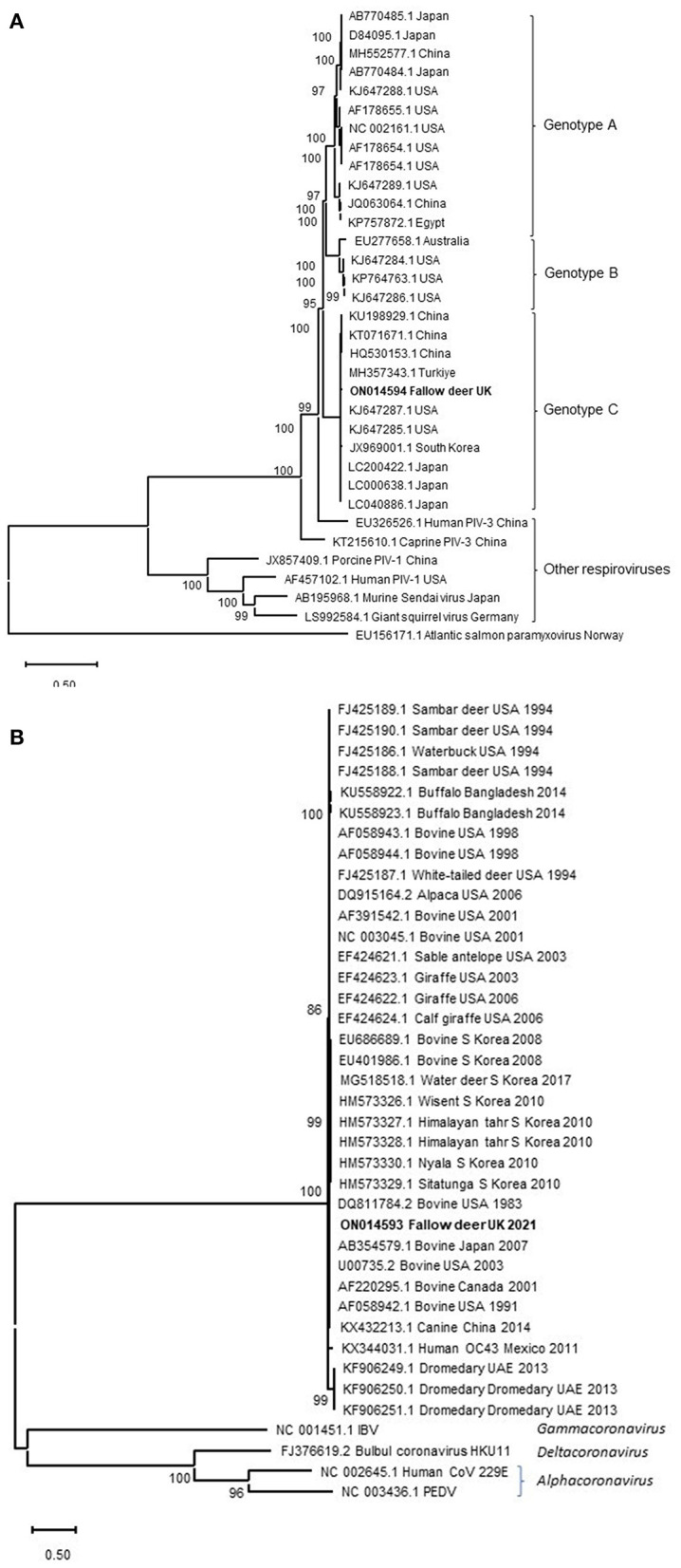
Maximum-likelihood phylogenetic analysis of PIV-3 L protein **(A)** and CoVs spike protein **(B)** from the fallow deer applying Neighbor-Join algorithm. The tree with the highest log likelihood (−75,708.09) is shown. Representative viruses in the *Respirovirus* genus are included in the PIV-3 phylogenetic tree. Atlantic salmon paramyxovirus in the *Aquaparamyxovirus* genus was used an outgroup. Animal CoVs in the *Betacoronavirus* genus and representatives of other CoVs genera are included in the CoVs' phylogenetic tree. A discrete Gamma distribution was used to model evolutionary rate differences among sites [5 categories (+G, parameter = 0.7913)]. The tree is drawn to scale, with branch lengths measured in the number of substitutions per site. The percentage of trees in which the associated taxa clustered together in the bootstrap analysis is shown next to the branches. Bootstrap values <50% were omitted.

Following the findings of histopathology and IHC for PIV-3, NGS was performed to characterize the suspected viral pathogen further. NGS resulted in 8,821,420 sequence reads from which 34,779 (0.39%) reads were assembled to generate near complete PIV-3 (15,583 nucleotides) genome. In addition, 5,420 (0.061%) of the reads have composed a near complete CoV genome (30,976 nucleotides). Average length of sequence reads was 123 and 137 bases, with a median coverage of 495.22 and 26.77 for PIV-3 and CoV, respectively. These genome sequences were deposited in GenBank under accession numbers ON014594 (PIV-3) and ON014593 (CoV). The PIV-3 showed the highest sequence identity for the L protein gene to those of BPIV-3 (83.1–98.4%) in the *Respirovirus* genus. Phylogenetically, the virus clustered with PIV-3 reported from cattle in China, USA, south Korea and Turkiye in the genotype C (Figure 2A). No L protein gene sequence was available for the PIV-3 from Argentina to be included in the phylogenetic tree. The CoV spike protein gene shared 96.7% to 97.9% sequence identity with those of bovine CoVs in the *Betacoronavirus* genus, which is in line with phylogenetic analysis (Figure 2B). This identity with NCBI SARS-CoV-2 reference virus sequence (accession number NC_045512.2) was only at 53.1%. Following the detection of CoV RNA by NGS, albeit at much lower viral load compared to PIV-3, IHC for BCoV also revealed limited labeling of a low number of individual cells in the tracheal mucosa.

## Discussion

Here we investigated the cause of death in a fallow deer calf suffering from loss of body condition, diarrhea, malaise and dyspnoea. Pathological and virological investigations revealed PIV-3 to be the primary agent causing acute respiratory disease, with characteristic pneumonic pathology. The pneumonia was complicated further by bacterial and parasitic infections. A CoV was also detected in the lung tissue at a much lower load (~one sixth of PIV-3, based on the proportion of sequence reads), but it could not be directly linked to the pathology of the lung.

The calf had a moderately heavy gastrointestinal worm burden which possibly contributed to the poor condition of this calf and ill-thrift in the herd more widely. The enteric and pulmonary parasite infestations were likely acquired whilst at pasture, whereas the viral respiratory infections were acute and acquired most likely after housing, a well-recognized risk factor for respiratory disease in livestock. Stress and nutritional changes associated with weaning and housing were other potential contributory factors to the clinical picture. *E coli* was isolated in systemic distribution. This may have been a terminal infection in an animal otherwise debilitated by pneumonia and endoparasitism.

The two viruses detected were genetically closely related to those of known bovine PIV-3 and CoV. Housing the deer calves in the same airspace as heifers may have facilitated transmission of the viruses from the heifers. However, lack of PIV-3 and CoV RNA sequences from the heifers or UK cattle for comparison precludes verification of this transmission. Furthermore, PIV-3 antigens and antibodies have been detected in clinically normal deer, which may indicate deer-borne viruses. A study in Wisconsin detected antibodies to PIV-3 in 24.7% of the deer tested (38). Other research articles have also described presence of antibodies to PIV-3 in several deer species (39–45).

The Veterinary Deer Society (https://bds.org.uk/) had not received, at the time of this submission, reports of evidence of disease associated with PIV-3 infection in British deer but was of the opinion that any increase in intensification of deer farming, or increased mixing with other domestic ruminants may change the situation (Aiden Foster, personal communication). Therefore, this report linking PIV-3 with typical pathology and disease in British deer is evidence to support that notion.

Spillover of SARS-CoV-2 to deer populations has been of particular concern in North America. From 283 retropharyngeal lymph node (RPLN) samples collected from free-living and captive white-tailed deer in Iowa from April 2020 through January of 2021, 33.2% were positive for SARS-CoV-2 RNA (26). The November 2020 peak of human cases in Iowa, which was also coinciding with the onset of winter and the peak deer hunting season, has contributed to an even higher prevalence of infection; 80 of 97 RPLN samples (82.5%) collected over a 7-week period were positive for SARS-CoV-2 RNA. The potential for deer species as reservoirs and a source of SARS-CoV-2 evolution and subsequent spill back to humans may have unpredictable health and welfare consequences for both humans and deer species. Therefore, respiratory and enteric disease cases in deer species merit thorough investigation.

Overall, this case highlights the challenges potentially arising from intensification of farming, co-housing of species and other management practices that increase the risk of pathogen spill-over and disease expression. It also highlights the value of an integrated investigative approach, combining traditional and advanced pathological and virological techniques in investigation of animal diseases. Further research could be aimed at both exploring wider knowledge of viral pathogens of deer, and their potential links to viral infections of other ruminants. These studies may also shed further light on cross-species transmission of the viruses between deer and other species if co-farmed.

## Data availability statement

The datasets presented in this study can be found in online repositories. The names of the repository/repositories and accession number(s) can be found below: https://www.ncbi.nlm.nih.gov/genbank/, ON014593 and ON014594.

## Author contributions

AD, TF, and VS: conceptualization, writing–original draft preparation, and supervision. AD, TF, HD, VS, and AB: acquisition of data. AD, TF, HD, and VS: interpretation of data. AD, TF, VS, HD, AB, and AW: writing–review and editing. All authors have read and agreed to the published version of the manuscript.

## Funding

This work was funded by the Department of Environment, Food and Rural Affairs and the Welsh Government through the Scanning Surveillance for Disease in Miscellaneous and Exotic farmed species and Cattle in England and Wales (ED1500 and ED1000) projects at APHA and EJPOH COVRIN https://onehealthejp.eu/jip-covrin/. One Health EJP which has received funding from the European Union's Horizon 2020 research and innovation programme under grant agreement no. 773830 ©2018.

## Conflict of interest

The authors declare that the research was conducted in the absence of any commercial or financial relationships that could be construed as a potential conflict of interest.

## Publisher's note

All claims expressed in this article are solely those of the authors and do not necessarily represent those of their affiliated organizations, or those of the publisher, the editors and the reviewers. Any product that may be evaluated in this article, or claim that may be made by its manufacturer, is not guaranteed or endorsed by the publisher.
